# A systematic review/meta‐analysis of prevalence and incidence rates illustrates systemic underrepresentation of individuals racialized as Asian and/or Asian‐American in ADRD research

**DOI:** 10.1002/alz.13820

**Published:** 2024-05-06

**Authors:** Yiqi Zhu, Soobin Park, Ramana Kolady, Wenqing Zha, Ying Ma, Amanda Dias, Katherine McGuire, Angela Hardi, Sunny Lin, Zahinoor Ismail, Paris B. Adkins‐Jackson, Jean‐Francois Trani, Ganesh M. Babulal

**Affiliations:** ^1^ School of Social Work Adelphi University Garden City New York USA; ^2^ Brown School Washington University in St. Louis St. Louis Missouri USA; ^3^ University of Rochester Rochester New York USA; ^4^ Department of Neurology Washington University School of Medicine St. Louis Missouri USA; ^5^ University of Houston 56B M.D. Anderson Library Houston Texas USA; ^6^ Bernard Becker Medical Library Washington University School of Medicine St. Louis Missouri USA; ^7^ Division of General Medical Sciences Department of Medicine Washington University School of Medicine St. Louis Missouri USA; ^8^ Departments of Psychiatry Clinical Neurosciences, and Community Health Sciences Hotchkiss Brain Institute University of Calgary Calgary Alberta Canada; ^9^ Department of Clinical and Biomedical Sciences Faculty of Health and Life Sciences University of Exeter Devon UK; ^10^ Departments of Epidemiology and Sociomedical Sciences Mailman School of Public Health Columbia University New York New York USA; ^11^ Institute of Public Health Washington University St. Louis Missouri USA; ^12^ Centre for Social Development in Africa Faculty of Humanities University of Johannesburg Cnr Kingsway & University Roads Johannesburg South Africa; ^13^ National Conservatory of Arts and Crafts Paris France; ^14^ Department of Clinical Research and Leadership The George Washington University School of Medicine and Health Sciences Washington District of Columbia USA

**Keywords:** Alzheimer's disease and related dementia, asian, asian‐american, disparity, incidence, prevalence

## Abstract

We investigate Alzheimer's disease and related dementia (ADRD) prevalence, incidence rate, and risk factors in individuals racialized as Asian and/or Asian‐American and assess sample representation. Prevalence, incidence rate, risk factors, and heterogeneity of samples were assessed. Random‐effects meta‐analysis was conducted, generating pooled estimates. Of 920 records across 14 databases, 45 studies were included. Individuals racialized as Asian and/or Asian‐American were mainly from Eastern and Southern Asia, had higher education, and constituted a smaller sample relative to non‐Hispanic white cohorts. The average prevalence was 10.9%, ranging from 0.4% to 46%. The average incidence rate was 20.03 (12.01‐33.8) per 1000 person‐years with a range of 75.19–13.59 (12.89‐14.33). Risk factors included physiological, genetic, psychological, behavioral, and social factors. This review underscores the systemic underrepresentation of individuals racialized as Asian and/or Asian‐American in ADRD research and the need for inclusive approaches accounting for culture, language, and immigration status.

**Highlights:**

- There is considerable heterogeneity in the prevalence of ADRD among studies of Asian‐Americans.
- There is limited data on group‐specific risk factors for ADRD among Asian–Americans.
- The average prevalence of (ADRD) among Asian–Americans was found to be 7.4%, with a wide range from 0.5% to 46%.

## 1 INTRODUCTION

Dementia prevalence will experience exponential growth worldwide over the next three decades.^1^ Alzheimer's disease (AD) is the most common dementia etiology, followed by vascular, Lewy body, frontotemporal, and Parkinson's disease. In 2023, 6.7 million Americans ≥ 65 years of age (an older adult) were living with AD in the United States (US), equating to roughly 1 in 9 people (10.7%) in this age band.^2^ AD is expected to rise to 12.7 million by 2050, almost doubling from the 2023 estimate.^2^ Racial and ethnic disparities in dementia incidence are pervasive and embedded in structural and social determinants of health (S/SDOH).^3^, ^4^ Unsurprisingly, AD disparity is influenced by S/SDOH such as inequitable education attainment, fewer financial resources and limited healthcare access, which stem from institutional racism and discrimination established by historical policies.^2^, ^4^, ^5^, ^6^ Institutional racism extends to scientific practices that aggregate distinct national and cultural groups into racialized categories that reflect external perceptions of commonalities. Existing dementia studies often aggregate data for individuals racialized as Asian, Asian‐American, and Native Hawaiian/Pacific Islander into a single group, thereby masking intragroup disparities.^7^, ^8^, ^9^ The aggregation of diverse groups of people into an “Asian” category results in findings that suggest such a group has a decreased risk for dementia than other groups, which may lead to biases that have significant ramifications for certain groups and individuals.^7^

Characterizing an individual as “Asian” and/or “Asian‐American”, is a part of racialization, a process that assigns a category and value to individuals based on perceived similarities.^10^ This label is a social construct that is highly contextual and subject to change based on time and space. “Asian” refers to a nebulous racialized category used to denote the identity of diverse peoples that currently constitute 60% of the global population and represent over 2,300 distinct languages. In the United States, “Asian” is used as a racialized category by the Office of Management and Budget (OMB) and Census to distinguish individuals of perceived or self‐reported Asian ancestry, including people from the Far East, Southeast Asia, and the Indian subcontinents while excluding persons from the Middle East, West Asia, and the Caucasus region.^11^ This arbitrary categorization diminishes the complexity of nationality, history, and cultural backgrounds. Interestingly, this racialization is assigned to groups irrespective of country of origin as future generations of “Asian” descendants are racialized similarly to someone born in an “Asian” country. On occasion, these individuals may be referred to as “Asian‐American” to acknowledge their affiliation with the United States and to demarcate their connection to the Asian continent. Despite individuals racialized as Asian and/or Asian‐American in the United States being considered a smaller portion of the population, they represent one of the fastest‐growing racialized groups in the United States, and older adults in this group are projected to grow 145% by 2030.^12^ Globally, the number of individuals racialized as Asian (based on OMB definition) is projected to grow to 5.25 billion by 2055.^13^ However, individuals racialized as Asians in the United States often experience exclusion by being grouped into a monolith in scientific research obfuscating an understanding of which specific risk and protective factors impact the incidence and prevalence of Alzheimer's disease and related dementia (ADRD).

**RESEARCH IN CONTEXT**

1. **Systematic review**. Empirical peer‐reviewed studies that were published in the United States after 1990 were included. The sampling methods and heterogeneity of included articles were assessed using meta‐analysis, generating pooled estimates.
2. **Interpretation**. Samples with individuals racialized as Asian and/or Asian‐American tend to have smaller sizes with higher education, mainly from Eastern and Southern Asia. High heterogeneity existed among those articles. The average prevalence was 7.4% ranging from 0.5% to 46%. The average incidence was 20.03 (12.01‐33.8) with a range of 75.19–13.59 (12.89, 14.33) per 1000 person‐year. The findings exposes the underrepresentation of individuals racialized as Asian and/or Asian‐American in ADRD studies, heterogeneity in prevalence and incidence measurement, and gaps in understanding group‐specific risk factors.
3. **Future directions**. The review recommends future studies collect data on nationality, generational status, and linguistic proficiency, disaggregate samples by country of origin, immigration status, and structural and social determinants of health, and enhance reporting of language usage during assessments to foster more inclusive research with individuals racialized as Asian and/or Asian‐American.

The history of individuals racialized as Asian and/or Asian‐Americans in the United States is riddled with residential segregation, workplace discrimination, and food insecurity.^14^, ^15^, ^16^ When integrated with individuals racialized as White, individuals racialized as Asian and/or Asian‐American experience a myriad of interpersonal discrimination, including microaggressions, some leading to more rapid cognitive decline.^17^ Most notably, the perceived socioeconomic and integration successes of a few have fueled the construction of the “model minority myth” that erroneously posits that individuals racialized as Asian and/or Asian‐American have achieved socioeconomic and social success, yielding benefits to health and well‐being in the United States. Therefore, these individuals are not underrepresented, do not experience health disparities, and should be excluded from social and healthcare policy reforms.^18^ This myth has led to a lack of standards of care for individuals racialized as Asian and/or Asian‐American, resulting in systemic underestimation of ADRD prevalence. Of the limited studies that include individuals racialized as Asian and/or Asian‐American, estimates of AD or ADRD prevalence are often inconsistent due to variations in data collection of race and ethnicity, cognitive and clinical assessments used, and statistical methods reported. For example, factors such as the inclusion of resident location (community vs. institution), ^19^ choices of clinical assessments or neurological batteries used for dementia diagnosis (many of which are not accessible for people who speak an “Asian” language), ^7^ or specifying an Asian nationality under consideration^19^ all contribute to the uncertainty of prevalence rates among individuals racialized as Asian and/or Asian‐American. There is an underestimation of the various etiologies ensconced under the umbrella of ADRD, which are not mutually exclusive, as mixed dementia tends to be more prevalent in minoritized groups compared to older adults racialized as non‐Hispanic White (nHW) individuals.^20^, ^21^

Given these limitations in understanding the prevalence and potential risk factors for ADRD among older adults racialized as Asian and/or Asian‐American, these populations face multiple risks, including underdetection, ^22^ delayed diagnosis, ^7^, ^19^ and suboptimal management of cognitive impairment. For example, a recent study of racial and ethnic differences in ADRD diagnosis rates found that Medicare beneficiaries racialized as Asian and/or Asian‐American received fewer diagnostic evaluations for dementia compared to beneficiaries racialized as nHW individuals.^23^ A growing body of literature is finding that individuals racialized as Asian and/or Asian‐American face significant barriers to healthcare access including language and communication issues, inequitable cultural competency, and less/poorer insurance coverage.^24^, ^25^ Furthermore, the spotlight is shifting to the role of immigration as a risk factor for ADRD. Given that immigrants from Asia make up the largest immigrant group in the United States, ^26^ it is imperative to develop a better understanding of the prevalence and barriers to ADRD diagnosis for individuals racialized as Asian and/or Asian‐American in the United States, including recent immigrants. There is a *sine qua non* requirement in studies to compare a minoritized group to individuals racialized as nHW individuals; the control/gold‐standard group is used as a benchmark for rigor in scientific design. Regardless of the classification system codifying a group, there is significant heterogeneity within groups that needs to be recognized as essential, especially for minoritized groups.

With this context in mind, we conducted a systematic and meta‐analytical review with three primary objectives: (1) estimate the prevalence and incidence rate of dementia among individuals racialized as Asian and/or Asian‐American, and compare the prevalence and incidence rate to individuals racialized as nHW individuals in the United States, (2) ascertain whether adults racialized as Asian and/or Asian‐American are adequately represented in dementia research, and (3) conduct an analysis on the risk factors associated with dementia for adults racialized as Asian and/or Asian‐American identified in the existing literature.

## 2 METHODS

### 2.1 Literature Search Strategy

The published literature was systematically searched on October 18, 2022, using strategies developed by a medical librarian and authors (A.H., Y.Z., G.M.B.) to identify relevant articles pertaining to dementia, race and ethnicity, and individuals racialized as Asian and/or Asian‐American. To adopt a rigorous, methodological approach, the systematic review followed the PICO framework, with the population of interest centering on individuals racialized as Asian and/or Asian‐American residing in the United States. This selection was made due to the distinct racial and ethnic classifications used in the United States. Only articles published and available from January 1, 1990, to October 18, 2022, that reported the prevalence or incidence rate of ADRD or its risk factors among any population racialized as Asian and/or Asian‐American were included in the review. Notably, mild cognitive impairment (MCI) was excluded from this review given the heterogeneity in etiology, and it is not uniformly considered a formal ADRD diagnosis. The focus of this review targeted empirical peer‐reviewed studies that have specific ADRD diagnoses. Theoretical articles, systematic reviews, and published abstracts were excluded from this review. The search strategies were created using a combination of controlled vocabulary terms and keywords and were executed in Embase.com, Ovid–Medline All, Cochrane Library, Scopus, and Web of Science from database inception. All database searches were completed on October 18, 2022. Complete search strategies are provided in the supplementary material.

### 2.2 Inclusion and exclusion criteria

This systematic review encompassed all empirical studies focusing on ADRD among individuals racialized as Asian and/or Asian‐American residing in the United States. Studies must have a precise measure of ADRD or diagnostics criteria to be included. Articles were excluded if (1) the study was conducted outside of the United States, (2) the study did not report the prevalence or incidence rate of ADRD within a sample racialized as Asian and/or Asian‐American, (3) the study was published prior to 1990, or (4) the study was not a peer‐reviewed empirical investigation (e.g., published abstracts, commentaries, position papers, or letters to the editor).

### 2.3 Data Extraction and Synthesis

Search results were uploaded to Covidence^27^ for further screening. Two reviewer teams (W.Z. and R.K. or A.D. and K.M.) conducted a review of the title and abstract, following the inclusion and exclusion criteria described earlier. Any conflicts that arose were resolved through discussion and reconciliation by a third reviewer (S.P., M.Y., Y.Z., or G.M.B.). After the title and abstract screening, a similar process was applied to the full‐text reviews to confirm the relevance of the remaining articles. The included articles were then organized into three categories: (1) articles reporting the prevalence or incidence rate of ADRD, (2) articles focusing on the risk factors associated with ADRD, and (3) articles reporting the prevalence and incidence rate *and* conducted an analysis on the risk factors.

A total of 920 records were retrieved from the database literature search and then imported into Endnote's reference management software. There were 402 duplicate citations removed using a technique described by Bramer et al. (2018), ^28^ and one more duplicate citation was identified manually and removed. After removing duplicates, 517 unique citations remained for analysis and were uploaded to Covidence for screening. Two more articles from the references search were identified during the article review process. After screening all 519 article titles and abstracts to check for their relevance to our search aim, only 156 articles remained in our sample for a full‐text review. There were 111 articles further excluded after the full‐text review due to (1) a lack of reported details about which ADRD measures were used in the study, (2) missing information about ADRD in a sample racialized as Asian and/or Asian‐American, and (3) the study not being conducted in the United States.

Key information was extracted from the included articles: title, authors, publication year, funding sources, aims and objectives, study design, study inclusion and exclusion criteria, total sample size, and ADRD measurement. To analyze the representation of adults racialized as Asian and/or Asian‐American in ADRD research, the definition of Asian and/or Asian‐American used in the study, the sample size, and demographic characteristics (such as age, sex, gender, education, income) were extracted from each study.

To obtain prevalence and incidence rates for the meta‐analysis, the number of ADRD cases, incidences, and person‐years for both groups racialized as Asian and/or Asian‐American and comparison groups were extracted from the datasets used in the studies. In the event that multiple studies utilized the same dataset/cohort study, the prevalence or incidence rate was extracted from the articles specifically reporting on either rate or both for that study.

To analyze the risk factors associated with ADRD among adults racialized as Asian and/or Asian‐American, the identified risk factors (explanatory or dependent variables) that showed significant associations with ADRD in each study were extracted. These factors were then categorized into physiological, genetic, psychological, behavioral, and social categories.

### 2.4 Statistical Analysis

Following data abstraction, the heterogeneity between studies was assessed using the Cochran Q statistic, and its magnitude was quantified using the I^2^ statistic. An I^2^ value greater than 50% indicates significant heterogeneity.^23^ In the presence of heterogeneity, random effects meta‐analysis was conducted to obtain pooled estimates of the prevalence and incidence rate of ADRD. Forest plots were utilized to visually represent the heterogeneity between different studies and display the pooled estimates. The pooled estimates and forest plots were performed using R version 4.3 using the Metafor package.^29^

## 3 RESULTS

**FIGURE 1:**
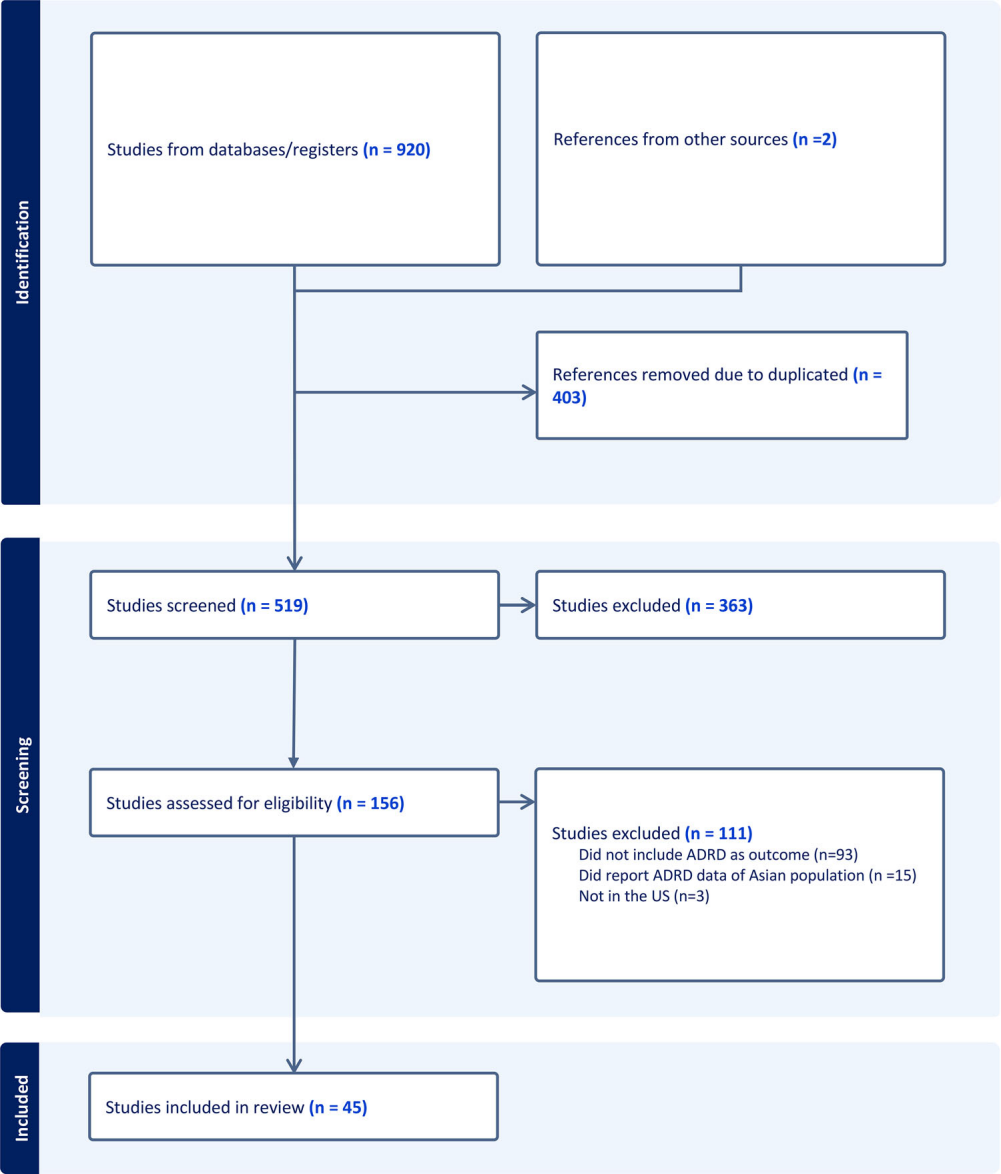
PRISMA flow diagram of studies screened and included in this systematic review.

There were 45 publications that examined data from fourteen unique datasets (Figure 1). Among the 45 studies, 26 studies used ADRD incidence rate as an outcome, which described the development of ADRD over a specific period, and 17 studies used ADRD prevalence, which reported the occurrence of ADRD at one‐time point. One study reported incidence diagnosis and one study reported both prevalence and incidence rate.

### 3.1 Individuals racialized as Asian and/or Asian‐American

Out of 45 studies, 22 (48.9%) used the Honolulu–Asia Aging Study cohort. Six studies used data from Kaiser Permanente Northern California healthcare members who participated in the California Men's Health Survey and Kaiser Permanente Research Program on Genes, Environment, and Health. Two studies used data from The KAME project (not an acronym) in Seattle, Washington, and the remaining studies used other cohorts nationwide or community samples with ADRD data.

A summary of the demographic characteristics of samples racialized as Asian and/or Asian‐American included in these datasets is provided in Table 1. Among these studies, 15 utilized datasets incorporating ADRD data from multiple racial and ethnic groups^7^, ^22^, ^23^, ^30^, ^31^, ^32^, ^33^, ^34^, ^35^, ^36^, ^37^, ^38^, ^39^, ^40^ nationwide or in certain regions. These datasets include Medicare Fee‐for‐Service claim data, ^22^ National Nursing Home Survey, ^31^ Multiethnic Cohort Study, ^8^ National Alzheimer's Coordinating Center, ^30^ Veterans Health Administration, ^36^ Women Health Initiatives (WHI)^31^ and Kaiser Permanente Northern California.^7^, ^33^, ^34^, ^37^, ^38^, ^39^ Three small‐scale studies^40^, ^41^, ^42^ recruited participants from multiple racial and ethnic groups from institutions or communities.

**TABLE 1:** Key demographics of Asian or Asian‐American participants included across the 45 studies.

Studies	Datasets	Asian group	Asian sample size (% of total participants)	Age M (SD)	Gender (% female)	Education (average years and SD or % high school and above)	Others/comments
Babulal et al. (2022)	National Alzheimer's Coordinating Center Uniform Data Set	Asian	186(2.73%)	73.61(5.71)	63.98%	89.01%	Asian participants have significantly higher years of education than nHW individuals
Kornblith et al. (2022)	Veterans Health Administration	Asian	9391(0.5%)	70.4(9.0)	5.4%	NA	Asian participants have higher percentage of women participants than other race and ethnicities and highest likelihood to live in zip code area with more than 25% of people having college education.
Tsoy et al. (2021)^a^	Medicare claim data in California	Asian	219, 908 (12%)	82.97(7.7)	64.8%	NA	
Lim et al. (2021)	Multiethnic cohort study based on Medicare claim data	Japanese American	32432(30.6%)	71.00(4.8)	54%	97%	The average age of Japanese American is significantly older than Filipino and whites.
		Filipino	4694(4.43%)	69.6(4.2)	53%	85%	Filipino had a significantly higher percentage of college graduate than Japanese American, but lower than whites.
Blue et al. (2021)	The women health initiative observational study and randomized clinical Trials	Asian	222(2%)	NA	100%	NA	NA
Fan et al. (2019)	Memory and Aging Center at University of California, San Francisco	Chinese	48(51%)	69(8.5)	52.1%	14.9(3.3)	There was not a significant difference between Chinese and White individuals in the demographic variables
Li et al. (2019)	Alzheimer's Disease Research Center pilot study at Icahn School of Medicine at Mount Sanai	Chinese American	122 (100%)	73.9(7.02)	69%	12.75(4.41)	
Matthew et al. (2019)	Medicare Fee for Service beneficiaries	API	694, 419(0.25%)	NA	NA	NA	
Davis et al. (2005)	National Nursing Home Survey	API	10, 356(6.65%)^b^	83.35(10.084)	100%	NA	
Huang et al. (2003)	Admission Minimal datasets	Chinese	125(47.89%)	82.3(8.9)	57.6%	NA	Compared with White individuals, they were more likely to be married, less likely to have lived alone, more likely to be using Medicaid, less likely to make medical decision alone, and more likely to depend on family members for decision‐making.
Silverman et al. (1992)	New York City nursing home	Chinese	1982 (55.72%)	NA	NA 46.7%	NA	
Hayes‐Larson et al. (2023) (2021), Mobley et al. (2021), Mayeda et al. (2017), (2016), (2014)^c^	KPNC^d^	Chinese	8384 (3.06%)	71.9	48.3%	41.8%	Asians were younger, had lower average income, and were less likely to live alone. Japanese Americans were older and had higher income. Asian Americans were represented in both highest and lowest educational attainment category.
		Filipino	6210(2.26%)	71.4	54%	59.9%	
		Japanese	4478(1.6%)	72.4	63.9%	49.8%	
		South Asian	197 (0.1%)	69.8	26.9%	50.7%	
		Others/unknown Asians	3763(1.6%)	71	50.7%	34.2%	
Borenstein et al. (2014) Graves et al. (1996)	KAME	Japanese American	1985(100%)	73.0(7.1)	57%	12(3)	
White et al. (1996) and other 18 studies^e^	The Honolulu‐Asia Aging Study	Japanese American	3734 (100%)	78	0%	45%	

^a^ Based on the Research Triangle Institute (RTI; based on an algorithm using beneficiaries’ surnames to identify race/ethnicity).

^b^ Based on the weighted sample.

^c^ The demographic information in based on Mayeda et al. (2016) and (2017).

^d^ Kaiser Permanente Northern California health plan members who completed one of two harmonized health surveys: California Men's Health Study or Kaiser Permanente Research Program on Genes, Environment, and Health Survey.

^e^ The rest of seven studies were based on Honolulu–Asia Aging Study Autopsy data.

Operationalization of race and ethnicity varied across studies. Of 15 studies that focused on racial comparisons^7^, ^8^, ^22^, ^23^, ^30^, ^31^, ^32^, ^33^, ^34^, ^35^, ^36^, ^37^, ^38^, ^39^, ^40^, ^41^, ^42^ four studies^7^, ^22^, ^30^, ^31^ used the OMB six‐category self‐reported ethnoracial groups. Kornblith et al. (2022)^36^ only used five categories, which did not distinguish between Hispanic and non‐Hispanic regarding groups racialized as Black and White. Multiethnic Cohort Study^8^ and Kaiser Permanente Northern California^7^, ^33^, ^34^, ^37^, ^38^, ^39^ collected country of origin to further classify participants racialized as Asian and/or Asian‐American. The study by Tsoy et al. (2021) used the Research Triangle Institute's algorithm to identify race/ethnicity based on the surnames of participants.^23^ The remaining studies relied on community partners to assist with participants’ racial and ethnic identity; however, most studies did not utilize nationality for their definition. Only Graves et al. (1996)^43^ specifically delineated Japanese Americans in the KAME project as a person with at least 50% of their ancestors identifying as being from Japan. Since different studies used varying terminology to describe race and ethnicity, the terminology reported in this section reflects how the original studies operationalized Asian and/or Asian‐American as a racialized group.

There were notable grouping decisions concerning the representation of Asian participants in these datasets. First, Matthews et al. (2019)^22^ used Medicare Fee‐for‐Service claim dataset and Davis et al. (2005)^32^ used National Nursing Home Survey grouped Asian and/or Asian‐American together with those racialized as Pacific Islander, forming an Asian American and Pacific Islander (AAPI) category. Second, the percentage of AAPI participants only accounted for 2% of the overall sample. In the remaining datasets, AAPI populations were reported separately, describing low Asian representation ranging from 2.73% in Babulal et al. (2022) using the National Alzheimer's Coordinating Center dataset^30^ to 0.5% in Kornblith et al. (2022) based on the Veterans Health Administration dataset.^36^

Representation was relatively higher in regional large‐scale datasets like Kaiser Permanente Northern California and Multiethnic Cohort Study since they are based in California, where populations racialized as Asian and/or Asian‐American represented 17% of the population in 2022.^44^ Asian representation ranged from 8.6% in Tsoy et al. (2021), ^23^ which is based on California Medicare fee‐for‐service beneficiaries enrolled in 2013‐2015, to 35% in Lim et al. (2022).^8^ However, among samples racialized as Asian and/or Asian‐American in Kaiser Permanente Northern California and Multiethnic Cohort Study, participants who immigrated from China, Japan, and the Philippines, accounted for over 50% of Asian and/or Asian‐American, while less than 5% of participants identified as immigrants or descendants of ancestors from the rest of Asian countries combined.^8^, ^33^, ^37^ Three studies in New York City and one in San Francisco centered on the experiences of Chinese Americans. Of these studies, two recruited participants from nursing homes, ^35^, ^40^ and the other two studies recruited patients from medical schools.^41^ Finally, the Honolulu‐Asia Aging Study cohort located in Honolulu, Hawaii, and The Kame Project located in Seattle, Washington State, recruited Japanese American patients.^12^, ^43^

### 3.2 ADRD Assessment

The operationalization of ADRD varied among the 45 studies (Table 2). Among studies which collected primary data, Fan et al. (2019)^41^ and Li et al. (2019)^42^ used criteria outlined by McKhann et al. (1984)^45^ and (2011)^45^, ^46^ after conducting a cognitive assessment battery adopted for the National Alzheimer's Coordinating Center Uniform Data Set (UDS).^47^ Graves et al. (1996)^12^ and White et al. (1996)^48^ employed the Diagnostic and Statistical Manual of Mental Disorders (DSM) IV^49^ and III‐Revised^50^ in both KAME project and Honolulu–Asia Aging Study cohort after administering the cognitive functioning screen, behavioral, and neurological examination. The Honolulu–Asia Aging Study also adopted Cummings and Benson (1992)^51^ criteria. White et al. (1996)^48^ further incorporated CT scans and blood tests (complete blood cell count, chemistry profile, vitamin B_12_ level, folate level, rapid plasma reagin, and thyroid function tests). Additionally, White et al. (1996)^48^ also used the autopsy criteria for comparison in the Honolulu–Asia Aging Study cohort. Silverman et al. (1992)^40^ was the only study that utilized the Alzheimer's Disease Risk Questionnaire^52^ to obtain information on ADRD.

**TABLE 2:** Operationalization of ADRD.

Measures	Studies
ICD‐9 and ICD‐10	Hayes‐Larson (2023), Kornblith et al. (2022), Lim et al. (2021), Mobley et al. (2021), Tsoy et al. (2021), Matthew et al. (2018), Hayes‐Larson (2021), Mayeda et al. (2017), Mayeda et al. (2016), Mayade et al. (2014), Davis et al.(2005),
Mckann et al. (1984); Mckann et al. (2011)	Fan et al (2019), Li et al. (2019);
DSM	Borenstein et a. (2014), Graves et al. (1996), White et al. (1996)
CDR > 0.5	Babulal et al. (2022)
CDR > 1	Graves et al. (1996)
Cummings and Benson (1992)	White et al. (1996), Graves et al. (1996)
Self reported medical history	Blue et al. (2021), Huang et al. (2003), Silverman et al. (1992)
Neuropathologic criteria (Autopsy data)	Jorm et al. (2004), Petrovich (2001), Petrovich et al. (2004), Launer et al (2008), Peila et al. (2007). Petrovich et al. (2008), White et al. (2005)

Studies based on Medicare and Kaiser Permanente Northern California used ICD codes to identify patients with ADRD. While National Alzheimer's Coordinating Center data includes various ADRD diagnoses of participants, these data are dependent on their follow‐up duration, where more follow‐up allows for better characterization. The diagnostic tests in the National Alzheimer's Coordinating Center are not validated for different groups racialized as Asian and/or Asian‐American. For example, Ng et al. (2006) found that the Mini–Mental Status Examination (MMSE) has significantly lower specificity and sensitivity among Malay people in Singapore compared to Chinese and Indian people. They also found that the MMSE performs worse among groups with lower access to education, which may disproportionately and systematically impact certain individuals racialized as Asian and/or Asian‐American.^53^

### 3.3 Meta‐analysis of the prevalence and incidence of the ADRD

Sixteen studies reported the prevalence of ADRD among different groups racialized as Asian and/or Asian‐American based on cross‐sectional datasets (Table 3). Six of the studies^48^, ^54^, ^55^, ^56^, ^57^, ^58^ used the Honolulu–Asia Aging Study, where only two reported the specific dementia etiology. Graves et al. (1996)^43^ focused on AD, multi‐infarct dementia, other dementia, and undetermined dementia etiology, while White et al. (1996)^48^ focused on probable and possible AD, vascular dementia, mixed dementia, other dementia, or undermined dementia cause. Li et al. (2019)^42^ and Davis (2005)^32^ examined AD etiology and all causes of dementia (or ADRD) separately. Fan et al. (2019)^41^ solely reported on the prevalence of AD and the rest of the studies reported the prevalence of general ADRD. Due to the small number of studies differentiating a specific form of dementia, the meta‐analysis only compared the prevalence of ADRD across different studies. The prevalence of each study was reported in Table 3.

**TABLE 3:** Prevalence of Alzheimer's disease and related dementia among Asian participants.

Study	Racial category	Measures	Prevalence of ADRD and 95% CI
Blue et al. (2021)	API	Medical history	0.072 [0.042‐ 0.114]
Fan et al. (2019)	Chinese	McKhann et al. (1984)	0.167 [0.075‐0.302]
Li et al. (2019)	Chinese	McKhann et al. (2011)	0.133 [0.078‐0.207]
Matthew et al. (2019)	API	ICD‐9‐CM	0.101 [0.100‐0.102]
Davis (2005)	API	ICD‐9	0.460 [0.450‐0.470]
Huang et al. (2003)	Chinese	Health History	0.336 [0.254‐0.426]
Graves et al. (1996)	Japanese American	DSM‐III‐R and CDR > 1	0.054 [0.044‐0.065]
White et al. (1996)^*^	Japanese American	DSM‐III‐R	0.061 [0.053‐0.069]
Silverman et al. (1992)	Chinese	Family history	0.008 [0.005‐0.013]

* The other six studies that are also based on the ADRD prevalence in White et al. (1996): Curb et al. (1999), Higuchi et al. (2015), Stewart et al. (2005), Launer et al. (2000), and Stewart et al. (2007).

**FIGURE 2:**
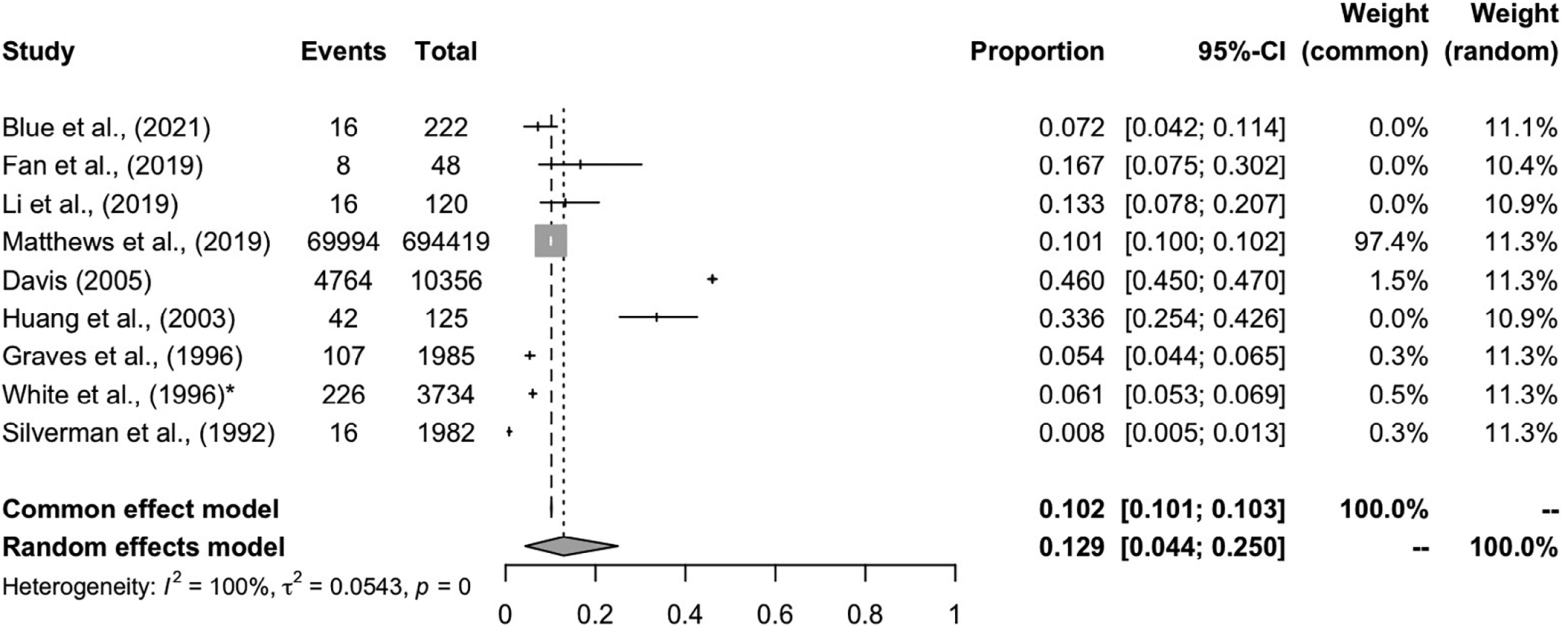
The forest plot of the prevalence of Alzheimer's disease and related dementia among Asian participants. ^*^The other six studies that are also based on the ADRD prevalence in White et al. (1996): Curb et al. (1999), Higuchi et al. (2015), Stewart et al. (2005), Launer et al. (2000), Stewart et al. (2007) and Gelber et al. (2012).

The average prevalence of ADRD across these studies was 12.9% (95% confidence interval [CI]: 4.4%−25%) (Figure 2). The highest prevalence (46%) was found by Davis (2005)^32^ via a nursing home survey. In contrast, Silverman et al. (1992) reported the lowest prevalence (0.8%) among the first‐degree relatives of the nondemented older adults living in senior centers in New York City.^40^

There was considerable heterogeneity across study samples(I^2 ^= 100%). Matthews et al. (2019)^22^ had the largest sample size of Private Fee‐for‐Service beneficiaries racialized as Asian and/or Asian‐American (n = 694, 419) from 2014. Studies employing community‐based recruitment with a focus on studying AD‐associated cortical atrophy, ^41^ cognitive tests, ^42^ and healthcare service utilization tend to have a higher proportion^32^ of participants diagnosed with ADRD.

Twenty‐two studies used the incidence rate from longitudinal studies of ADRD as the outcome variable (Table 4). Among these, 12 studies were based on the Honolulu–Asia Aging Study cohort.^56^, ^59^, ^60^, ^61^, ^62^, ^63^, ^64^, ^65^, ^66^, ^67^, ^68^, ^69^ We used the 3‐year incidence rate reported in Havlik et al. (2000)^63^ for the meta‐analysis. Six studies^7^, ^33^, ^34^, ^37^, ^38^, ^39^ based on the Kaiser Permanente Northern California dataset reported a similar incidence rate, so only the incidence ratio reported in Mayeda et al. (2016)^7^ was used in meta‐analysis. Among those studies, Borenstein et al. (2014)^12^ and Havlik et al. (2000)^63^ reported incidence rates of different dementia subtypes in the KAME project and Honolulu–Asia Aging Study^95^ cohort, respectively.

**TABLE 4:** Incidence rate of Alzheimer's disease and related dementia among Asian participants.

Study	Racial category	Measures	Incident rate (per 1000‐person year)
Babulal et al. (2022)	Asian	CDR > 0.5	75.19 [57.87‐97.7]]
Kornblith et al. (2022)	Asian	ICD‐9 and 10	13.59 [12.89‐14.33]
Lim et al. (2021)	Asian	ICD‐9 and 10	15.14 [14.75‐15.54]
Mayeda et al. (2016) ^*^	Asian American	ICD‐9	17.16 [16.63‐17.72]
Borenstein et al. (2014)	Japanese American	DSM‐IV	14.09 [12.09‐16.42]]
Havlik et al. (2000) ^**^	Japanese American	DSM‐III‐R	18.10 [15.31‐21.40]

* The other five studies that are based on the similar incident rate reported in Mayeda et al. (2016): Hayes–Larson et al. (2023), Hayes–Larson et al. (2022), Mobley et al. (2023), Mayeda et al. (2017) and Mayeda et al. (2014).

** The other 11 studies that are based on the similar incident rate reported in Havlik et al. (2000): Foley et al. (2001), Peila et al. (2004), Saczynski et al. (2006), Yucesoy et al. (2006), Laurin et al. (2007), Saczynski et al. (2007), Irie et al. (2008), Crane et al. (2009), de Jong et al. (2009), Kalmijn et al. (2000) and Gelber et al. (2011).

**FIGURE 3:**
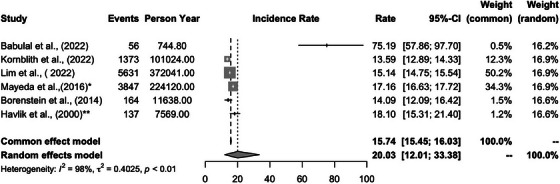
The forest plot of the incidence rate of Alzheimer's disease and related dementia among Asian participants. ^*^The other five studies that are based on the similar incident rate reported in Mayeda et al. (2016): Hayes–Larson et al. (2023), Hayes–Larson et al. (2022), Mobley et al. (2023), Mayeda et al. (2017) and Mayeda et al. (2014). ^**^The other 11 studies that are based on the similar incident rate reported in Havlik et al. (2000): Foley et al. (2001), Peila et al. (2004), Saczynski et al. (2006), Yucesoy et al. (2006), Laurin et al. (2007), Saczynski et al. (2007), Irie et al. (2008), Crane et al. (2009), de Jong et al. (2009), Kalmijn et al. (2000) and Gelber et al. (2011).

An I^2^ statistic of 98%, suggested that there was significant heterogeneity among the 17 studies included in our meta‐analysis. The study by Babulal et al. (2022)^30^ used National Alzheimer's Coordinating Center data with participants was an outlier from the rest since it reported a considerably higher incidence rate than the rest of the studies: 75.19 (95% CI: 57.19‐97.70) per 1000 person‐year (Figure 3). When Babulal et al. (2022)^30^ was included, the average incidence rate across was 20.03 (95% CI: 12.01‐33.8) per 1000 person‐year using random effects model. However, when the study was excluded, the incidence rate lowered to 15.34 (95% CI: 13.92‐17.13).

### 3.4 Ethnoracial group comparison

Three studies compared the difference in odds of ADRD between the Asian group and nHW individuals. Huang et al. (2003)^35^ and Matthews et al. (2019)^22^ reported lower odds in Asians than in nHW individuals. Tsoy et al. (2021)^23^ reported the incidence diagnosis of older Asian adults where 0.4% of Asian beneficiaries were dementia‐free from 2013 to 2014 but then received a dementia diagnosis in 2015 and had higher odds of being diagnosed with ADRD compared to nHW individuals. However, there was not a significant difference in the odds of a diagnosis of cognitive impairment in general (ADRD and MCI) among different ethnoracial groups. Four studies compared the hazard rate of ADRD between Asians and nHW individuals. Kornblith et al. (2022)^36^ reported a higher hazard for Asians compared to nHW individuals; two studies^7^, ^8^ reported a lower hazard rate for Asians for ADRD, and a fourth study^30^ did not find a statistically significant difference in hazard between Asians and nHW individuals.

### 3.5 Risk factors associated with ADRD

Twenty‐three studies reported a range of risk factors that were associated with ADRD beyond demographic factors (e.g., age, race, ethnicity).^31^, ^33^, ^34^, ^39^, ^54^, ^55^, ^56^, ^57^, ^58^, ^59^, ^60^, ^61^, ^62^, ^63^, ^64^, ^65^, ^66^, ^67^, ^68^, ^69^, ^71^, ^72^, ^73^, ^74^ The significant risk factors are organized in Table 5. Most of the studies (n = 19) investigating specific risk factors of ADRD among individuals racialized as Asian and/or Asian‐American were based in the Honolulu–Asia Aging Study cohort. Physiological factors and genetic factors were the most frequently studied factors, with 12 studies focused on them. Conversely, psychological, social, and behavioral factors were not as extensively explored, having been the focus of only eight studies.

**TABLE 5:** Putative Alzheimer's disease and related dementia risk factors.

Physiological risk factors	Impaired glucose tolerance	Curb et al. (1999)	Only significantly associated with vascular dementia
	Fasting insulin	Peila et al. (2004)	Two extremes of insulin distribution (lowest and highest 15^th^ percentile) had an increased risk for dementia.
	Thyroid function	deJong et al (2009)	Higher total and free thyroxine levels were associated with an increased risk of dementia.
	Declined cholesterol levels	Stewart et al. (2007)	Cholesterol levels in men with dementia and, in particular, those with Alzheimer disease had declined at least 15 years before the diagnosis and remained lower than cholesterol levels in men without dementia throughout that period.
	Metabolic Cardiovascular Syndrome	Kalmijin et al. (2000)	The Z‐score for random post‐load glucose, diastolic and systolic blood pressures, body mass index, subscapular skinfold thickness, random triglycerides, and total cholesterol. The Z‐score sum was higher in demented subjects than in nondemented subjects.
	Midlife blood pressure	Launer et al. (2000)	The risk of dementia was significantly associated with never/ever treated blood pressure, but not associated with treated men.
**Genetic factor**	MYH11, FZD3, and SORCS3	Blue et al. (2021)	The study identified significant associations between variants within APOE, MYH11, FZD3, SORCS3, and GOLGA8B and the risk of dementia. Ten genes implicated by these loci, including MYH11, FZD3, SORCS3, and GOLGA8B, were differentially expressed in the context of Alzheimer's disease.
	APOE‐e4	Havlik et al. (1999), Irie et al. (2008)	Men with an APOE4 allele had a significantly increased risk of AD after adjusting for age and education.
	single nucleotide polymorphisms (SNPs) in the interleukin‐1(IL‐1) family in a large	Yecesoy et al. (2006)	
	Serum levels of apolipoprotein A‐I	Saczynski (2007)	
	Ankle‐to‐brachial index	Laurin et al. (2007)	
**Psychological factors**	Depression and antidepressant use	Babulal et al. (2022); Irie et al. (2008)	Depression was significantly associated with the progression of dementia. Participants who had depression but not using antidepressants had a higher risk compared with those who had depression but used antidepressants.
**Behavioral factor**	Daytime Sleepiness	Foley et al. (2001)	
	Coffee Intake	Gelber et al. (2011)	There were no significant associations between coffee or caffeine intake and risk of cognitive impairment, overall dementia, AD, VaD, or moderate/high levels of the individual neuropathologic lesion types. However, men in the highest quartile of caffeine intake (> 277.5 mg/d) were less likely than men in the lowest quartile (≤115.5 mg) to have any of the lesion types (adjusted‐OR, 0.45; 95% CI, 0.23–0.89; p, trend = 0.04).
	Low education	Hayes‐Larson (2023)	
	Memory Complaints	Jorm et al. (2004)	Memory complaints were found to predict the neuropathologic diagnosis of AD after adjusting for age, time to death, education, depression, and cognitive functioning.
**Social factors**	Neighborhood disadvantage	Mobley et al. (2021)	
	Decreased social engagement	Saczynski et al. (2006)	

Studies have shown that immigration status and language may also be important risk factors for ADRD but are significantly understudied.^4^ Among the studies identified in this review, most studies were not able to collect information on the immigration status or primary language used by the participants. Hayes–Larson et al. (2022)^33^ found that ADRD incidence rate appeared higher among the foreign‐born Filipinos and Japanese, but not among Chinese and individuals racialized as nHW individuals. Another investigation^59^ found that there was not a significant association between written Japanese proficiency and ADRD.

### 3.6 Autopsy data and pathological assessment

Seven studies were based on autopsy data from the Honolulu–Asia Aging Study cohort.^72^, ^73^, ^74^, ^75^, ^76^, ^77^, ^78^ Neuropathological data was available in the Honolulu–Asia Aging Study for 58 male decedents. Results from the seven studies^72^, ^73^, ^74^, ^75^, ^76^, ^77^, ^78^ indicated three notable trends: (1) AD lesions characterized by neurofibrillary tangles and neuritic plaques were associated with hippocampal sclerosis (significant neuronal loss); (2) brain atrophy (summary of brain weight, mantle thickness, microscopic neuronal loss) was associated with more moderate/severe AD lesions; and (3) generalized brain atrophy was associated with more moderate/severe levels of microvascular infarcts. In addition, White et al. (2005)^77^ found a significant discordance between the clinical diagnosis and the neuropathological assessment of ADRD, further highlighting the complexity of ADRD clinical assessment.

## 4 DISCUSSION

To our knowledge, this systematic review and meta‐analysis is the first to examine the evidence from empirical studies, quantify heterogeneities of prevalence and incidence rate, and elucidate the challenges pertaining to the sample representation, diagnosis criteria, and risk factors across the studies involving older adults racialized as Asian and/or Asian‐American in the United States. Lim et al. (2020)^78^ conducted the first scoping review of ADRD in the AAPI population to map the extant literature. This systematic review and meta‐analysis further evaluates empirical evidence and found that the pooled effects estimated by random effects models were 12.90% (95% CI: 4.4%−25%) for prevalence and 20.03% (95% CI 12.01‐33.38) for incidence rates. There was significant heterogeneity (I^2^) due to the different inclusion criteria of Asian groups and measures of ADRD. Most research has concentrated on physiological and genetic factors as the principal risk factors. In contrast, psychological, behavioral, and social factors have not been explored as extensively. Additionally, pathology data in ADRD are often lacking for minoritized groups, especially for Asian Americans.^79^ The disparity between clinical and pathological diagnosis of ADRD was highlighted in seven studies included in the present review, but all the data used in these studies were generated by the Honolulu–Asia Aging Study cohort.

There is a profound gap in the ADRD literature on prevalence and incidence rates among United States adults racialized as Asian and/or Asian‐American.^8^ Only 45 studies were identified based on 13 unique datasets across the United States. Additionally, two thirds (30/45) of the studies were based on three projects: Honolulu‐Asian Aging Study, KAME, and Kaiser Permanente Northern California. While these three cohorts are well‐characterized, the findings cannot be generalized to individuals of the same ethnoracial group in different cities or individuals racialized as Asian in the overall United States. In retrospective studies using national datasets such as the National Alzheimer's Coordinating Center or Medicare claims data, participants racialized as Asian and/or as American make up less than 3% of the cohorts, and most community studies are only able to work with a small sample of participants from one given Asian group. The overall lack of diversity in the available data, coupled with a selection bias in each dataset, constitutes an important limitation in our capacity to effectively measure ADRD prevalence and incidence among adults racialized as Asian and/or Asian‐American.^79^, ^81^

Our findings suggest that aggregating diverse and heterogeneous populations into a monolithic “Asian” category masks significant differences between groups and among individuals.^81^ In regional datasets such as Multiethnic Cohort Study and Kaiser Permanente Northern California datasets, while the percentage of participants racialized as Asian and/or Asian‐American was much larger, the specific groups included were predominantly East Asian such as Chinese, Filipino, and Japanese. Participants from other distinct countries and regions were often excluded or lumped into the “other Asian” group, which may also mask important socio‐economic differences among populations in addition to cultural and political ones. In studies included in this systematic review, the average income and education level of study participants racialized as Asians were often significantly higher than those of participants racialized as nHW individuals, suggesting significant sample selection bias in existing datasets. This lack of representation may stem from overly simplistic data collection practices that predicate using convenience samples, a common problem also found with nHW cohorts in observational studies and clinical trials.^83^, ^84^, ^85^, ^86^ The Census continues to use the OMB classification, standardized in 1980, which consists of six (addition of ‘Other’) racialized categories defined by the dominant (European and/or European‐American) culture.^86^ Before 1980, the Social Security Administration only used three categories (White/Black/Other).^86^ As a result, racial identifiers in Medicare datasets and many other large datasets are generated using these broad, externally informed categories. However, subsequent studies have found that people identified as Hispanic and Asian are often miscategorized.^87^, ^88^

The overall practice of simplifying the data collection process was also reflected in the fact that few studies collected data on language and immigration status, including refugee status, which deeply influences screening, assessing, and diagnosing ADRD. Given that immigrants from Asia make up the largest immigrant group^26^ in the United States and minoritized groups with limited English‐language proficiency, this is a critical knowledge gap in our understanding of risk factors for ADRD. In 2019, limited English‐language proficiency was approximately 31% among individuals racialized as Asian and/or Asian‐American (40% for Chinese, Bangladeshi, Vietnamese, Nepalese, and Burmese) and 12% for individuals racialized as Native Hawaiian and Pacific Islander. Prior work found that limited English‐language proficiency is associated with a delayed diagnosis of ADRD, ^88^ as well as lower income, education, and access to health insurance.^89^ Research on immigrants racialized as Hispanic observed a higher prevalence of ADRD compared to native‐born individuals racialized as Hispanic.^78^ A recent systematic review in Australia found that refugees and asylum seekers had higher rates of depression, social isolation, physical inactivity, and diabetes (modifiable ADRD risk factors) compared to their Australian‐born counterparts. These studies shed light on the potential risk factors that might be observed in individuals racialized as Asian and/or Asian‐American, given similar exposures.^90^

The lack of a concerted effort to engage diverse groups in ADRD research leads to an underestimation of the needs of individuals racialized as Asian and/or Asian‐American, many at risk of ADRD and delayed or missed diagnosis. This issue is further exacerbated by the lack of data disaggregation in research among groups categorized as Asian and/or Asian‐American. For example, among Cambodian and Bangladeshi individuals in the United States, poverty rates are approximately 20%, compared to 12.6% in individuals racialized as Asian‐American. Similarly, while 75% of individuals racialized as Asian‐American hold a bachelor's degree or higher, only 18% of Burmese and Hmong individuals attained a bachelor's degree.^9^ Since poorer access to educational attainment and lower income are significantly associated with the faster progression of dementia, ^34^, ^39^ the lack of representation of individuals racialized as Asian and/or Asian‐American with lower education and/or income in ADRD studies introduces significant sample bias.

ADRD, as a concept and formal medical diagnosis with different etiologies, intersects with language, social and familial roles, community beliefs, experiences, and perceptions, which can result in individuals being stigmatized by their community.^3^, ^92^ However, those risk factors have not been thoroughly examined in the studies included in this systematic review and meta‐analysis. Related to language, dementia does not exist in the lexicon of many languages. For example, among Hmong Americans, dementia is more commonly related to chronic confusion or *“tem toob*,” which is a term that normalizes forgetting things as one ages.^92^ As a result, a formal medical diagnosis of dementia, associated screening/assessment, and treatment may not translate or have the same impact without the appropriate cultural context. More often, the term dementia can have a negative and stigmatizing connotation. The elders are often held in high regard in the family/community, and a diagnosis like dementia may be interpreted as labeling the person with a mental illness.^93^ There is a need for the family to protect/insulate their elders from perceived stigmatizing conditions that may undermine their value and respect, cultivated over decades.^93^ This requires care providers and researchers to be sensitized to the cultural values of a community, understand the heterogeneity among adults racialized as Asian and/or Asian‐American, and use skilled and culturally responsive approaches to care and communication.

There were several important limitations to this review. We excluded studies conducted on MCI among samples racialized as Asian and/or Asian‐American, which both limit the generalizability of study results and reduce the number of studies we reviewed on ADRD prevalence and incidence rate. Additionally, given that two‐thirds of the studies originated from three cohort studies, two on the West Coast and one not in the mainland United States, geographical and sociopolitical factors may shape access to healthcare and time to a diagnosis differently. Generalizability may be limited when examining the same ethnoracial group in different cities. While not a limitation, it is important to note that we focused exclusively on studies that examined the experience of individuals racialized as Asian and/or Asian‐American living in the United States. Also, the English language restriction and inclusion of literature published since 1990 may have excluded published work with additional incidence and prevalence rates.

This review reveals critical areas for future work. More studies are needed to comprehensively understand the unique needs of participants racialized as Asian/Asian‐American. Recruiting participants who are racialized as Asian/Asian‐American could be challenging due to the stigma associated with ADRD, lack of sufficient funding, and access to specific communities. Engaging older adults racialized as Asian/Asian‐American and advocating for representation in research requires tailored approaches. These may include conducting community outreach programs with healthcare experts from these diverse communities, providing language assistance (e.g., interpreters), developing validated and linguistically tailored assessments, offering free, culturally‐sensitive cognitive screening, and implementing brain health education to destigmatize ADRD.

When we collect data, disaggregated data are needed on the nationality, generational status, language preference, and immigration/refugee status of participants racialized as Asian and Asian‐American.^94^ These data will provide a more robust understanding of how these factors impact ADRD risk in the United States. Our study suggests that language plays a critical role in the screening, assessment, and diagnosis of ADRD. Among the studies in this review, the wide variation in prevalence and incidence rate of ADRD among participants racialized as Asian and/or Asian‐American suggests that a lack of validation of diagnostic instruments across different languages may limit research on ADRD. Additional research is necessary to improve the accuracy and validity of diagnostic tests among individuals racialized as Asian and/or Asian‐American in the United States. In addition, moving forward, studies should report on English‐language proficiency in the study sample, whether English or another language was used in the assessment (including translated instruments), and any obstacles present during data collection that may impact the validity of the results. Finally, the lack of data disaggregation among groups racialized as Asian and Asian‐American masks critical differences between groups. In addition to disaggregation, data collection efforts should include diverse categories for nationality and ethnicity, structural and social determinants of health (racism, capitalism, sexism), multidimensionality poverty, and recruitment location of the participants.

## 5 CONCLUSION

In conclusion, this systematic review and meta‐analysis sheds light on the gaps and challenges in ADRD research concerning individuals racialized as Asian and/or Asian‐American. The study emphasizes the need for more comprehensive and nuanced approaches to studying ADRD within this population. This review brings attention to limitations in sample representation, diagnostic criteria, and cultural considerations among older adults racialized as Asian and/or Asian‐American. It underscores the scarcity of comprehensive datasets, especially for groups racialized as non‐East Asian, and the impact of language, culture, and immigration on ADRD progression, diagnosis, and care. This review proposes that future research should collect data on citizenship, generational status, and linguistic proficiency and incorporate analyses that examine country of origin, immigration status, and structural and social determinants of health. Studies should also enhance reporting of language usage during assessments to foster more inclusive and nuanced ADRD research with individuals racialized as Asian and/or Asian‐American.

## CONFLICT OF INTEREST STATEMENT

All authors declare no conflicts related to this work. Author disclosures are available in the supporting information.

## Supporting information

Supporting Information

## References

[1] Wimo A , Seeher K , Cataldi R , et al. The worldwide costs of dementia in 2019. Alzheimer's & Dement. 2023. 10.1002/alz.12901 PMC10842637 36617519

[2] 2023 Alzheimer's disease facts and figures. Alzheimers Dement. 2023;19:1598‐1695. 36918389 10.1002/alz.13016

[3] Babulal GM , Quiroz YT , Albensi BC , et al. Perspectives on ethnic and racial disparities in Alzheimer's disease and related dementias: update and areas of immediate need. Alzheimer's & Dementia. 2019;15:292‐312. 10.1016/j.jalz.2018.09.009 PMC6368893 30555031

[4] Adkins‐Jackson PB , George KM , Besser LM , et al. The structural and social determinants of Alzheimer's disease related dementias. Alzheimers Dement. 2023. 10.1002/alz.13027 PMC10599200 37074203

[5] Chin AL , Negash S , Hamilton R . Diversity and disparity in dementia: the impact of ethnoracial differences in Alzheimer disease. Alzheimer Dis Assoc Disord. 2011;25:187‐195. 21399486 10.1097/WAD.0b013e318211c6c9 PMC3396146

[6] Mukadam N , Cooper C , Livingston G . Improving access to dementia services for people from minority ethnic groups. Curr Opin Psychiatry. 2013;26:409‐414. 23454888 10.1097/YCO.0b013e32835ee668 PMC4222802

[7] Mayeda ER , Glymour MM , Quesenberry CP , Whitmer RA . Inequalities in dementia incidence between six racial and ethnic groups over 14 years. Alzheimers Dement. 2016;12:216‐224. 26874595 10.1016/j.jalz.2015.12.007 PMC4969071

[8] Lim U , Wang S , Park SY , et al. Risk of Alzheimer's disease and related dementia by sex and race/ethnicity: the Multiethnic Cohort Study. Alzheimers Dement. 2022;18:1625‐1634. 34882963 10.1002/alz.12528 PMC9177893

[9] Budiman A , Ruiz NG , Key facts about Asian Americans, a diverse and growing population. 2021.

[10] Adkins‐Jackson PB , Rodriguez ACI . Methodological approaches for studying structural racism and its biopsychosocial impact on health. Nurs Outlook. 2022;70:725‐732. 36154771 10.1016/j.outlook.2022.07.008 PMC11298157

[11] Bureau USC . 2020 Census Frequently Asked Questions About Race and Ethnicity. 2021.

[12] Borenstein AR , Wu Y , Bowen JD , et al. Incidence rates of dementia, Alzheimer disease, and vascular dementia in the Japanese American population in Seattle, WA: the Kame Project. Alzheimer Dis Assoc Disord. 2014;28:23‐29. 24045327 10.1097/WAD.0b013e3182a2e32f PMC4036673

[13] Nation U . World Popluation Prospect 2022. 2022.

[14] Intrator J , Tannen J , Massey DS . Segregation by race and income in the United States 1970‐2010. Soc Sci Res. 2016;60:45‐60. 27712688 10.1016/j.ssresearch.2016.08.003 PMC5117629

[15] Islam JY , Awan I , Kapadia F . Food insecurity, financial hardship, and mental health among multiple Asian American ethnic groups: findings from the 2020 COVID‐19 household impact survey. Health Equity. 2022;6:435‐447. 35801150 10.1089/heq.2021.0179 PMC9257551

[16] Shang Z , Kim JY , Cheng SO . Discrimination experienced by Asian Canadian and Asian American health care workers during the COVID‐19 pandemic: a qualitative study. Can Med Assoc Open Access J. 2021;9:E998‐E1004. 10.9778/cmajo.20210090 PMC8598237 34785529

[17] Tang F , Li K , Rosso AL , Jiang Y , Li M . Neighborhood segregation, socioeconomic status, and cognitive function among older Chinese immigrants. J Am Geriatr Soc. 2023;71:916‐926. 36508718 10.1111/jgs.18167 PMC10023380

[18] Tendulkar SA , Hamilton RC , Chu C , et al. Investigating the myth of the “model minority”: a participatory community health assessment of Chinese and Vietnamese adults. J Immigr Minor Health. 2012;14:850‐857. 21874359 10.1007/s10903-011-9517-y

[19] Mehta KM , Yeo GW . Systematic review of dementia prevalence and incidence in United States race/ethnic populations. Alzheimers Dement. 2017;13:72‐83. 27599209 10.1016/j.jalz.2016.06.2360

[20] Barnes LL , Leurgans S , Aggarwal NT , et al. Mixed pathology is more likely in black than white decedents with Alzheimer dementia. Neurology. 2015;85:528‐534. 26180136 10.1212/WNL.0000000000001834 PMC4540250

[21] Filshtein TJ , Dugger BN , Jin L‐W , et al. Neuropathological diagnoses of demented Hispanic, black, and non‐Hispanic white decedents seen at an Alzheimer's disease center. Journal of Alzheimer's Disease. 2019;68:145‐158. 10.3233/JAD-180992 PMC7286069 30775996

[22] Matthews KA , Xu W , Gaglioti AH , et al. Racial and ethnic estimates of Alzheimer's disease and related dementias in the United States (2015‐2060) in adults aged >/= 65 years. Alzheimers Dement. 2019;15:17‐24. 30243772 10.1016/j.jalz.2018.06.3063 PMC6333531

[23] Tsoy E , Kiekhofer RE , Guterman EL , et al. Assessment of Racial/Ethnic Disparities in Timeliness and Comprehensiveness of Dementia Diagnosis in California. JAMA Neurol. 2021;78:657‐665. 33779684 10.1001/jamaneurol.2021.0399 PMC8008426

[24] Chen JA , Zhang E , Liu CH . Potential impact of COVID‐19–related racial discrimination on the health of Asian Americans. Am J Public Health. 2020;110:1624‐1627. 32941063 10.2105/AJPH.2020.305858 PMC7542280

[25] McMurtry CL , Findling MG , Casey LS , et al. Discrimination in the United States: experiences of Asian Americans. Health Serv Res. 2019;54:1419‐1430. 31657465 10.1111/1475-6773.13225 PMC6864377

[26] Radford J , Key findings about US immigrants. 2020.

[27] Innovation VH . Covidence systematic review software.

[28] Bramer WM , De Jonge GB , Rethlefsen ML , Mast F , Kleijnen J . A systematic approach to searching: an efficient and complete method to develop literature searches. J Med Libr Assoc: JMLA. 2018;106:531. 30271302 10.5195/jmla.2018.283 PMC6148622

[29] Viechtbauer W . Conducting meta‐analyses in R with the metafor package. J Stat Softw. 2010;36:1‐48.

[30] Babulal GM , Zhu Y , Roe CM , et al. The complex relationship between depression and progression to incident cognitive impairment across race and ethnicity. Alzheimers Dement. 2022;18:2593‐2602. 35213795 10.1002/alz.12631 PMC9402798

[31] Blue EE , Thornton TA , Kooperberg C , et al. Non‐coding variants in MYH11, FZD3, and SORCS3 are associated with dementia in women. Alzheimers Dement. 2021;17:215‐225. 32966694 10.1002/alz.12181 PMC7920533

[32] Davis JA . Differences in the health care needs and service utilization of women in nursing homes: comparison by race/ethnicity. J Women Aging. 2005;17:57‐71. 16186095 10.1300/J074v17n03_05

[33] Hayes‐Larson E , Fong J , Mobley TM , et al. The role of nativity in heterogeneous dementia incidence in a large cohort of three Asian American groups and white older adults in California. Alzheimers Dement. 2022;18:1580‐1585. 35103385 10.1002/alz.12563 PMC9339576

[34] Hayes‐Larson E , Ikesu R , Fong J , et al. Association of Education With Dementia Incidence Stratified by Ethnicity and Nativity in a Cohort of Older Asian American Individuals. JAMA Netw Open. 2023;6:e231661. 36877520 10.1001/jamanetworkopen.2023.1661 PMC9989900

[35] Huang ZB , Neufeld RR , Likourezos A , et al. Sociodemographic and Health Characteristics of Older Chinese on Admission to a Nursing Home: a Cross‐Racial/Ethnic Study. J Am Geriatr Soc. 2003;51:404‐409. 12588586 10.1046/j.1532-5415.2003.51116.x

[36] Kornblith E , Bahorik A , Boscardin WJ , Xia F , Barnes DE , Yaffe K . Association of Race and Ethnicity With Incidence of Dementia Among Older Adults. JAMA. 2022;327:1488‐1495. 35438728 10.1001/jama.2022.3550 PMC9020215

[37] Mayeda ER , Glymour MM , Quesenberry CPJr , Whitmer RA . Heterogeneity in 14‐year Dementia Incidence Between Asian American Subgroups. Alzheimer Dis Assoc Disord. 2017;31:181‐186. 28406845 10.1097/WAD.0000000000000189 PMC5568954

[38] Mayeda ER , Karter AJ , Huang ES , Moffet HH , Haan MN , Whitmer RA . Racial/ethnic differences in dementia risk among older type 2 diabetic patients: the diabetes and aging study. Diabetes Care. 2014;37:1009‐1015. 24271192 10.2337/dc13-0215 PMC3964496

[39] Mobley TM , Shaw C , Hayes‐Larson E , et al. Neighborhood disadvantage and dementia incidence in a cohort of Asian American and non‐Latino White older adults in Northern California. Alzheimers Dement. 2023;19:296‐306. 35388625 10.1002/alz.12660 PMC9535033

[40] Silverman JM , Li G , Schear S , et al. A cross‐cultural family history study of primary progressive dementia in relatives of nondemented elderly Chinese, Italians, Jews and Puerto Ricans. Acta Psychiatr Scand. 1992;85:211‐217. 1561893 10.1111/j.1600-0447.1992.tb08597.x

[41] Fan J , Tse M , Carr JS , et al. Alzheimer Disease‐associated Cortical Atrophy Does not Differ Between Chinese and Whites. Alzheimer Dis Assoc Disord. 2019;33:186‐193. 31094707 10.1097/WAD.0000000000000315 PMC6527333

[42] Li C , Neugroschl J , Zhu CW , et al. Cognitive test battery for evaluating elderly Chinese Americans. Int Psychogeriatr. 2019;31:505‐511. 30277186 10.1017/S1041610218001060 PMC6511071

[43] Graves AB , Larson E , Edland SD , et al. Prevalence of dementia and its subtypes in the Japanese American population of King County, Washington State: the Kame Project. Am J Epidemiol. 1996;144:760‐771. 8857825 10.1093/oxfordjournals.aje.a009000

[44] Bureau USC . QuickFacts California. 2022.

[45] McKhann G , Drachman D , Folstein M , Katzman R , Price D , Stadlan EM . Clinical diagnosis of Alzheimer's disease: report of the NINCDS‐ADRDA Work Group* under the auspices of Department of Health and Human Services Task Force on Alzheimer's Disease. Neurology. 1984;34:939‐944. 6610841 10.1212/wnl.34.7.939

[46] McKhann GM , Knopman DS , Chertkow H , et al. The diagnosis of dementia due to Alzheimer's disease: recommendations from the National Institute on Aging‐Alzheimer's Association workgroups on diagnostic guidelines for Alzheimer's disease. Alzheimer's & dementia. 2011;7:263‐269. 10.1016/j.jalz.2011.03.005 PMC3312024 21514250

[47] Weintraub S , Salmon D , Mercaldo N , et al. The Alzheimer's Disease Centers' Uniform Data Set (UDS): the neuropsychologic test battery. Alzheimer Dis Assoc Disord. 2009;23:91‐101. 19474567 10.1097/WAD.0b013e318191c7dd PMC2743984

[48] White L , Petrovitch H , Ross GW , et al. Prevalence of dementia in older Japanese‐American men in Hawaii: the Honolulu‐Asia aging study. JAMA. 1996;276:955‐960. 8805729

[49] American Psychiatric Association A, Association AP . Diagnostic and statistical manual of mental disorders: DSM‐IV. American Psychiatric Association Washington, DC;1994.

[50] Association AP . Diagnostic and Statistical Manual ofMental Disorders, 3rd ed., Revised. American Psychiatric Association;1987.

[51] Cummings JL , Benson DF , Dementia: A clinical approach: Butterworth‐Heinemann Medical;1992.

[52] Breitner JC , Folstein MF . Familial Alzheimer dementia: a prevalent disorder with specific clinical features. Psychol Med. 1984;14:63‐80. 6200894 10.1017/s0033291700003081

[53] Ng TP , Niti M , Chiam PC , Kua EH . Prevalence and correlates of functional disability in multiethnic elderly Singaporeans. J Am Geriatr Soc. 2006;54:21‐29. 16420194 10.1111/j.1532-5415.2005.00533.x

[54] Curb JD , Rodriguez BL , Abbott RD , et al. Longitudinal association of vascular and Alzheimer's dementias, diabetes, and glucose tolerance. Neurology. 1999;52:971‐975. 10102414 10.1212/wnl.52.5.971

[55] Higuchi M , Chen R , Abbott RD , et al. Mid‐life proteinuria and late‐life cognitive function and dementia in elderly men: the Honolulu‐Asia Aging Study. Alzheimer Dis Assoc Disord. 2015;29:200‐205. 25626635 10.1097/WAD.0000000000000082 PMC4514569

[56] Laurin D , Masaki KH , White LR , Launer LJ . Ankle‐to‐brachial index and dementia: the Honolulu‐Asia Aging Study. Circulation. 2007;116:2269‐2274. 17967779 10.1161/CIRCULATIONAHA.106.686477

[57] Stewart R , Masaki K , Xue Q‐L , et al. A 32‐year prospective study of change in body weight and incident dementia: the Honolulu‐Asia Aging Study. Arch Neurol. 2005;62:55‐60. 15642850 10.1001/archneur.62.1.55

[58] Stewart R , White LR , Xue Q‐L , Launer LJ . Twenty‐six–year change in total cholesterol levels and incident dementia: the Honolulu‐Asia Aging Study. Arch Neurol. 2007;64:103‐107. 17210816 10.1001/archneur.64.1.103

[59] Crane PK , Gibbons LE , Arani K , et al. Midlife use of written Japanese and protection from late life dementia. Epidemiology. 2009;20:766‐774. 19593152 10.1097/EDE.0b013e3181b09332 PMC3044600

[60] de Jong FJ , Masaki K , Chen H , et al. Thyroid function, the risk of dementia and neuropathologic changes: the Honolulu‐Asia aging study. Neurobiol Aging. 2009;30:600‐606. 17870208 10.1016/j.neurobiolaging.2007.07.019 PMC3147246

[61] Foley D , Monjan A , Masaki K , et al. Daytime sleepiness is associated with 3‐year incident dementia and cognitive decline in older Japanese‐American men. J Am Geriatr Soc. 2001;49:1628‐1632. 11843995 10.1046/j.1532-5415.2001.t01-1-49271.x

[62] Gelber RP , Petrovitch H , Masaki KH , Ross GW , White LR . Coffee intake in midlife and risk of dementia and its neuropathologic correlates. J Alzheimer's Disease. 2011;23:607‐615. 21157028 10.3233/JAD-2010-101428 PMC3731132

[63] Havlik R , Izmirlian G , Petrovitch H , et al. APOE‐ε4 predicts incident AD in Japanese‐American men: the Honolulu–Asia Aging Study. Neurology. 2000;54:1526‐1529. 10751272 10.1212/wnl.54.7.1526

[64] Irie F , Masaki KH , Petrovitch H , et al. Apolipoprotein E ε4 allele genotype and the effect of depressive symptoms on the risk of dementia in men: the Honolulu‐Asia aging study. Arch Gen Psychiatry. 2008;65:906‐912. 18678795 10.1001/archpsyc.65.8.906 PMC4791584

[65] Kalmijn S , Foley D , White L , et al. Metabolic cardiovascular syndrome and risk of dementia in Japanese‐American elderly men: the Honolulu‐Asia Aging Study. Arterioscler Thromb Vasc Biol. 2000;20:2255‐2260. 11031212 10.1161/01.atv.20.10.2255

[66] Peila R , Rodriguez BL , White LR , Launer LJ . Fasting insulin and incident dementia in an elderly population of Japanese‐American men. Neurology. 2004;63:228‐233. 15277613 10.1212/01.wnl.0000129989.28404.9b

[67] Saczynski JS , Pfeifer LA , Masaki K , et al. The effect of social engagement on incident dementia: the Honolulu‐Asia Aging Study. Am J Epidemiol. 2006;163:433‐440. 16410348 10.1093/aje/kwj061

[68] Saczynski JS , White L , Peila RL , Rodriguez BL , Launer LJ . The relation between apolipoprotein A‐I and dementia: the Honolulu‐Asia aging study. Am J Epidemiol. 2007;165:985‐992. 17298957 10.1093/aje/kwm027

[69] Yucesoy B , Peila R , White LR , et al. Association of interleukin‐1 gene polymorphisms with dementia in a community‐based sample: the Honolulu‐Asia Aging Study. Neurobiol Aging. 2006;27:211‐217. 16226351 10.1016/j.neurobiolaging.2005.01.013

[70] Launer, Lenore J., et al. Midlife blood pressure and dementia: the Honolulu–Asia aging study☆. Neurobiol Aging. 2000;21(1):49‐55. 10794848 10.1016/s0197-4580(00)00096-8

[71] Gelber RP , Petrovitch H , Masaki KH , et al. Lifestyle and the risk of dementia in Japanese‐American men. J Am Geriatr Soc. 2012;60:118‐123. 22211390 10.1111/j.1532-5415.2011.03768.x PMC3258374

[72] Peila R , Yucesoy B , White LR , et al. A TGF‐beta1 polymorphism association with dementia and neuropathologies: the HAAS. Neurobiol Aging. 2007;28:1367‐1373. 16904244 10.1016/j.neurobiolaging.2006.06.004

[73] Petrovitch H , Ross GW , He Q , et al. Characterization of Japanese‐American men with a single neocortical AD lesion type. Neurobiol Aging. 2008;29:1448‐1455. 17499884 10.1016/j.neurobiolaging.2007.03.026 PMC2613368

[74] Petrovitch H , Ross GW , Steinhorn SC , et al. AD lesions and infarcts in demented and non‐demented Japanese‐American men. Ann Neurol. 2005;57:98‐103. 15562458 10.1002/ana.20318

[75] Jorm AF , Masaki KH , Davis D , et al. Memory complaints in nondemented men predict future pathologic diagnosis of Alzheimer disease. Neurology. 2004;63:1960‐1961. 15557525 10.1212/01.wnl.0000144348.70643.f2

[76] Launer LJ , Petrovitch H , Ross GW , Markesbery W , White LR . AD brain pathology: vascular origins? Results from the HAAS autopsy study. Neurobiol Aging. 2008;29:1587‐1590. 17466414 10.1016/j.neurobiolaging.2007.03.008 PMC3437222

[77] Petrovitch H , White L , Ross G , et al. Accuracy of clinical criteria for AD in the Honolulu–Asia Aging Study, a population‐based study. Neurology. 2001;57:226‐234. 11468306 10.1212/wnl.57.2.226

[78] White L , Small BJ , Petrovitch H , et al. Recent clinical‐pathologic research on the causes of dementia in late life: update from the Honolulu‐Asia Aging Study. J Geriatr Psychiatry Neurol. 2005;18:224‐227. 16306244 10.1177/0891988705281872

[79] Lim S , Mohaimin S , Min D , et al. Alzheimer's Disease and its Related Dementias among Asian Americans, Native Hawaiians, and Pacific Islanders: a Scoping Review. J Alzheimers Dis. 2020;77:523‐537. 32675416 10.3233/JAD-200509 PMC8638681

[80] Huie EZ , Whitmer RA , George KM , Dugger BN . Neuropathology studies of dementia in US persons other than non‐hispanic whites. Free neuropathology. 2022;3. 10.17879/freeneuropathology-2022-3795 PMC9007571 35425946

[81] Lim AC , Barnes LL , Weissberger GH , et al. Quantification of race/ethnicity representation in Alzheimer's disease neuroimaging research in the USA: a systematic review. Commun Med (Lond). 2023;3:101. 37491471 10.1038/s43856-023-00333-6 PMC10368705

[82] Holland AT , Palaniappan LP . Problems with the collection and interpretation of Asian‐American health data: omission, aggregation, and extrapolation. Ann Epidemiol. 2012;22:397‐405. 22625997 10.1016/j.annepidem.2012.04.001 PMC4324759

[83] Canevelli M , Bruno G , Vico C , et al. Socioeconomic disparities in clinical trials on Alzheimer's disease: a systematic review. Eur J Neurol. 2018;25:626‐e43. 10.1111/ene.13587 29383812

[84] Gleason CE , Norton D , Zuelsdorff M , et al. Association between enrollment factors and incident cognitive impairment in Blacks and Whites: data from the Alzheimer's Disease Center. Alzheimer's & Dementia. 2019;15:1533‐1545. 10.1016/j.jalz.2019.07.015 PMC6925619 31601516

[85] Keohane LM , Nikpay S , Braun K , et al. Association of race and income with incident diagnosis of Alzheimer's disease and related dementias among Black and White older adults. J Appl Gerontol. 2023;42:898‐908. 36469682 10.1177/07334648221142851 PMC10081951

[86] Llibre‐Guerra JJ , Heavener A , Brucki SMD , et al. A call for clinical trial globalization in Alzheimer's disease and related dementia. Alzheimer's & Dementia. 2023. 10.1002/alz.12995 PMC10450094 36840622

[87] Filice CE , Joynt KE . Examining race and ethnicity information in Medicare administrative data. Med Care. 2017;55:e170‐e176. 29135782 10.1097/MLR.0000000000000608

[88] Waldo DR . Accuracy and bias of race ethnicity codes in the Medicare enrollment database. Health Care Financ Rev. 2004;26:61‐72. 25371985 PMC4194866

[89] Low L‐F , McGrath M , Swaffer K , Brodaty H . Communicating a diagnosis of dementia: a systematic mixed studies review of attitudes and practices of health practitioners. Dementia. 2019;18:2856‐2905. 29544345 10.1177/1471301218761911

[90] Kovaleva M , Jones A , Maxwell CA . Immigrants and dementia: literature update. Geriatr Nurs (Minneap). 2021;42:1218‐1221. 10.1016/j.gerinurse.2021.04.019 34090727

[91] Hamrah MS , Bartlett L , Jang S , Roccati E , Vickers JC . Modifiable risk factors for dementia among migrants, refugees and asylum seekers in Australia: a systematic review. J Immigr Minor Health. 2023:1‐20. 10.1007/s10903-022-01445-2 PMC10212820 36652152

[92] Adkins‐Jackson PB , George KM , Besser LM , et al. The structural and social determinants of Alzheimer's disease related dementias. Alzheimer's & Dementia. 2023. 10.1002/alz.13027 PMC10599200 37074203

[93] Gerdner L , Tripp‐Reimer T , Yang D . Perception and care of elder Hmong Americans with chronic confusion or tem toob. Hallym Int J Aging. 2008;10:111‐138.

[94] Liu D , Hinton L , Tran C , Hinton D , Barker JC . Reexamining the relationships among dementia, stigma, and aging in immigrant Chinese and Vietnamese family caregivers. J Cross Cult Gerontol. 2008;23:283‐299. 18665444 10.1007/s10823-008-9075-5 PMC2958058

[95] Ismail Z , Babulal GM . Attitudinal adjustment about dementia awareness and assessment: finetuning inclusion, diversity, and measurement of behavioral and psychological symptoms. Int Psychogeriatr. 2023;35:7‐10. 36193702 10.1017/S1041610222000886 PMC9905302

